# Atypical Frontotemporal Connectivity of Cognitive Empathy in Male Adolescents With Conduct Disorder

**DOI:** 10.3389/fpsyg.2018.02778

**Published:** 2019-01-11

**Authors:** Daifeng Dong, Yali Jiang, Yidian Gao, Qingsen Ming, Xiang Wang, Shuqiao Yao

**Affiliations:** ^1^Medical Psychological Center, The Second Xiangya Hospital of Central South University, Changsha, China; ^2^Medical Psychological Institute of Central South University, Changsha, China; ^3^China National Clinical Research Center on Mental Disorders (Xiangya), Changsha, China; ^4^Department of Psychiatry, The First Affiliated Hospital of Soochow University, Suzhou, China

**Keywords:** conduct disorder, cognitive empathy, affective empathy, functional connectivity, adolescent

## Abstract

**Background:** It has been suggested that adolescents with conduct disorder (CD) may have a deficit in the affective and cognitive domains empathy, but studies exploring networks within the key brain regions of affective and cognitive empathy in adolescents with CD are lacking.

**Methods:** Functional connectivity (FC) analyses among key brain regions of the affective and cognitive empathy with resting-state functional magnetic resonance imaging (fMRI) were conducted in 30 adolescent boys with CD and 33 demographically matched healthy controls (HCs).

**Results:** Atypical FC within the key brain regions of affective empathy was not observed in CD adolescents. However, we found that CD adolescents showed decreased frontotemporal connectivity within the key brain regions of cognitive empathy in relation to HCs, that is, the FCs between right temporoparietal junction and ventromedial prefrontal cortex as well as dorsomedial prefrontal cortex.

**Conclusion:** These findings may provide insight into neural mechanism underlying a cognitive empathy deficiency of CD adolescents from the perspective of FC.

## Introduction

Conduct disorder (CD) is defined as a repetitive and persistent pattern of antisocial behavior in which the basic rights of others or social norms are violated (APA, 2013). CD has been reported to occur in about 16% of preadolescents (Olsson, 2009; Jiang et al., 2015). It has been suggested that antisocial behavior displayed by children with CD might be a result of atypical empathic responses to others’ suffering (Blair, 2005). The ability to empathize is critical for navigating complex social interactions and developing meaningful interpersonal relationships (Hooker et al., 2008; De Waal, 2012; Zaki and Ochsner, 2012).

Empathy, which refers to one’s cognitive as well as the emotional reactions to the observed experience of others (Shamay-Tsoory, 2011), is divided into affective and cognitive domains (Shamay-Tsoory et al., 2009; Shamay-Tsoory, 2011, 2013). More specifically, the capacity to experience affective reactions to the observed experiences of others or share a “fellow feeling” has been described as affective empathy. Its underlying processes include emotional contagion, emotion recognition and affect sharing (Shamay-Tsoory, 2011). The capacity to engage in the cognitive process of adopting another’s psychological point of view has been described as cognitive empathy, and its underlying processes include perspective taking and a more rational understanding of the emotions of others (Shamay-Tsoory, 2011). The neural substrate of affective empathy is thought to include anterior insula (AI), anterior cingulate cortex (ACC), and inferior frontal gyrus (IFG); while the neural substrate of cognitive empathy is thought to include the medial prefrontal cortex (mPFC), temporo-parietal junction (TPJ) and superior temporal sulcus (STS; Shamay-Tsoory, 2011, 2013; Walter, 2012; Raz et al., 2014).

A number of studies demonstrated that CD patients had an empathy deficit (Cohen and Strayer, 1996; Wied et al., 2005; Lovett and Sheffield, 2007; Schwenck et al., 2012). Relative to control subjects, individuals with CD have been shown to exhibit atypical empathic neural responses in brain areas associated with both affective and cognitive empathy, including mainly the amygdala (Sterzer et al., 2005; Stadler et al., 2007; Decety et al., 2009; Jones et al., 2009), AI (Lockwood et al., 2013; Michalska et al., 2015), ACC (Stadler et al., 2007; Lockwood et al., 2013), IFG (Lockwood et al., 2013), and TPJ (Decety et al., 2009; Dong et al., 2016). Thus, such findings are consistent with the suggestion that CD patients may be deficient in both domains of empathy. To our knowledge, although the brain regions associated with affective and cognitive empathy have been identified, studies exploring empathy domains in CD adolescents from the perspective of functional connectivity (FC) are lacking.

If CD adolescents have aberrant interactions within the key brain regions associated with cognitive and affective empathy, such alterations may be reflected in resting-state functional magnetic resonance imaging (fMRI) studies of FC, namely studies examining connectivity patterns in brains in the absence of task demands. Resting-state FC analysis can clarify the neural basis of specific behaviors or symptoms as a complement of task-fMRI approaches (Abram et al., 2016). Resting-state connectivity, which reveal temporal interactions between proximal and distal regions (Biswal et al., 1995), has been shown to predict individual differences in neural activity induced by the task (Tavor et al., 2016). Besides, resting-state FC data can also be used to examine the neural interaction associated with mental processes (Abram et al., 2016). Moreover, the resting-state findings are not been interfered by the individual variations including the attention, effort, or comprehension, thereby making resting-state analysis an effective tool of overcoming such limitations in task-based studies (Abram et al., 2016). Given these advantages, resting-state FC analysis provides an effective way by which to investigate the neural substrate underlying empathy.

With the aim of exploring whether there were altered FCs within the key brain regions of cognitive and affective empathy in male CD adolescents in comparison with the healthy controls (HCs), we conducted a rs-fMRI study with region of interest (ROI)-based FC analysis in 30 male CD adolescents and 33 demographically matched HCs. Based on previous studies, we hypothesized that CD adolescents would exhibit altered affective and cognitive empathy network connectivity.

## Materials and Methods

### Participants

The CD group consisted of 30 male adolescents who were recruited from out-patient clinics affiliated with the Second Xiangya Hospital of Central South University (Changsha, Hunan, China). The HCs included 34 healthy age-, and IQ-matched boys recruited from local middle schools in the same region. The Chinese version of the Wechsler Intelligence Scale for Children (C-WISC; Gong and Cai, 1993) was applied to measure IQ. The present study was approved by each school’s administration and the Ethics Committee of the Second Xiangya Hospital of Central South University. All participants and their parents were informed of the purpose of this study and provided written informed consent to be involved in the study.

The Structured Clinical Interview for the DSM-IV-TR Axis I Disorders-Patient Edition (SCID-I/P) (First et al., 2001) was administered to all participants by two well-trained psychiatrists. If there are inconsistence between the two psychiatrists, final decision will be made by the major researcher. All participants in the CD group were confirmed to fulfill the DSM-IV-TR criteria for CD (APA, 2000). Information was collected from each patient and at least one corresponding parent to improve the reliability of the diagnostic interview. A psychiatrist made the final decision as to whether the information provided by each patient and his parents were consistent.

None of the HCs met the criteria for psychiatric disorders, or had a history of CD symptoms or aggression. Both the CD adolescents and HCs were excluded from the study if they reported any following exclusion criteria: a history of attention deficit-hyperactivity disorder, oppositional-defiant disorder, or any psychiatric or emotional disorder; diagnosis of any pervasive developmental or chronic neurological disorder, Tourette syndrome, post-traumatic stress disorder, or obsessive compulsive disorder; persistent headaches; head trauma; a history of alcohol or substance abuse in the past year; contraindications to magnetic imaging; or an IQ ≤ 80.

### Clinical Assessments

Affective and cognitive empathy were evaluated with the Interpersonal Reactivity Index (IRI; Davis, 1980, 1983), which includes four subscales: empathic concern, perspective taking, fantasy, and personal distress. To assess the cognitive empathy we used the mean score of the perspective taking and fantasy subscales, whereas emotional empathy was assessed using the mean score of the empathic concern and personal distress subscales (Shamay-Tsoory et al., 2009). Callous unemotional (CU) trait phenotype was evaluated with the CU subscale of the Antisocial Process Screening Device (APSD; Frick and Hare, 2001; Vitacco et al., 2003). Six items were included in the callous unemotional subscale: cares about schoolwork; emotions are fake; feel bad when do something wrong; acts charming to get things; concerned about others’ feelings; hides feelings from others. The conduct problems subscale of the Strength and Difficulties Questionnaire (SDQ) was used to measure conduct problems of adolescents (Yao et al., 2009).

### Data Acquisition

Imaging data were acquired on a PHILIPS Achieva 3.0-T magnetic resonance scanner at the Second Xiangya Hospital of Central South University. All participants were instructed to lie in a supine position with their eyes closed, to remain still, and to think of nothing in particular, but to avoid falling asleep. Their heads were fixed snugly with foam pads to minimize head movement. Images were acquired with an echo planar imaging sequence with the following parameters: 36 axial slices, repetition time/echo time = 2000/30 ms, 64 × 64 matrix, 90° flip angle, field of view = 240 mm × 240 mm, thickness/gap = 4.0/0 mm, and 206 volumes. The total time of resting acquisition was 6 min 52 s.

### Data Processing

Image processing was performed in Data Processing Assistant for Resting-state fMRI program [DPARSFA V2.3, Chao-Gan and Yu-Feng (2010)^1^] and the Resting-state fMRI Data Analysis Toolkit [REST V1.8, Song et al. (2011)]. The following steps were included: (1) the first 10 volumes were discarded to allow for signal equilibration and adaptation of participants to scanning noise; (2) slice timing with the 18th slice as a reference slice; (3) head motion correction; (4) head motion scrubbing regressor (threshold for ‘bad’ time point, frame-wise displacements > 0.5 mm as well as 1 back and 2 forward neighbors); (5) spatial normalization based on echo-planar-imaging templates and resampling (3 mm × 3 mm × 3 mm); (6) smoothing with a 6-mm full-width at half maximum Gaussian kernel; (7) de-trending and filtering data with residual signals within 0.01–0.1 Hz to discard biases from high-frequency physiological noise and low-frequency drift.

The criterion for excessive head motion criterion was translation > 2 mm in any direction or rotation > 2° around any axis in six head motion parameters. One HC subject was excluded for excessive head motion.

### Statistical Analyses

#### Behavior

In the SPSS 18.0.0, independent two-sample *t*-tests were used to compare the distributions of age, IQ, and psychological profiles between the CD and HC groups.

#### Functional Connectivity

**FIGURE 1:**
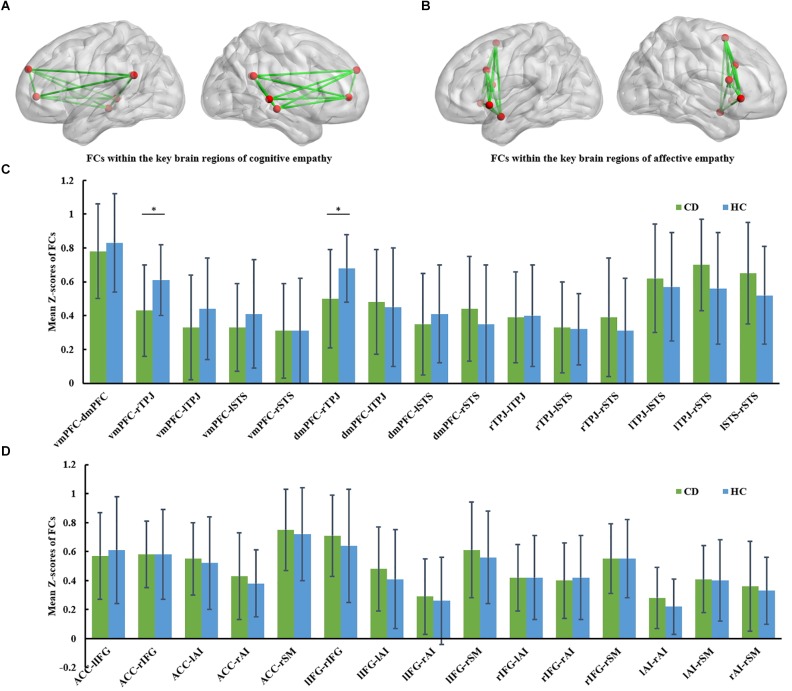
**(A)** FCs within the key brain regions of cognitive empathy. The cognitive empathy has 6 key brain regions including the ventromedial prefrontal cortex, dorsomedial prefrontal cortex, temporoparietal junction and superior temporal sulcus. The red spheres represent network nodes. The green edges represent inter-nodal functional connectivity (FC). **(B)** FCs within the key brain regions of affective empathy. The affective empathy has 6 key brain regions including the anterior cingulate, anterior insula, inferior frontal cortex and supplementary motor area. The red spheres represent network nodes. **(C)** Group differences between the conduct disorder (CD) group and healthy control (HC) group within the key brain regions of cognitive empathy. The CD group showed significantly reduced rTPJ-vmPFC and rTPJ-dmPFC FC relative to the HC group. ^∗^*FDR* corrected, *p* < 0.05. HC, healthy controls; CD, conduct disorder; vmPFC, ventromedial prefrontal cortex, dmPFC; dorsomedial prefrontal cortex; TPJ, temporoparietal junction; STS, superior temporal sulcus. **(D)** Group differences between the conduct disorder (CD) group and healthy control (HC) group within the key brain regions of affective empathy. No significant differences were observed between the CD group and HC group in the affective empathy network. ACC, anterior cingulate cortex; AI, anterior insula; IFG, inferior frontal cortex; SM, supplementary motor cortex.

Region of interest-based FC analyses were completed in REST software. The ROIs for cognitive empathy encompassed the vmPFC, dmPFC, TPJ, and STS; and the ROIs for affective empathy encompassed the AI, ACC, IFG and supplementary motor area. A total of 12 ROIs (6, cognitive empathy, Figure 1A; 6, affective empathy, Figure 1B) were defined with a sphere with a 3-mm radius. To be specific, the definition of ROIs mainly referred the article of Raz et al. (2014), which investigated the two modes of empathy by analyzing fMRI fluctuations of network cohesion with a ROI method. The 6 ROIs of cognitive empathy in our study were defined on the basis of a fMRI item analysis in a theory of mind task (Dodell-Feder et al., 2011), which was consistent with Raz et al. (2014). The 4 ROIs of affective empathy were defined on the basis of a meta-analysis on empathy for pain (Lamm et al., 2011), which was consistent with Raz et al. (2014). Besides, two additional ROIs (right IFG and supplementary motor area) of affective empathy were defined on the basis of fMRI meta-analysis of Fan et al. (2011). The right IFG and supplementary motor area are also important in affective empathy as the theory proposed by Shamay-Tsoory (2011). Detailed descriptions of the ROIs are provided in Supplementary Table S1.

We used the FC tool in REST software to extract correlation coefficients of each ROI pair; these values reflect the degree of temporal connectivity between ROI pairs (Wisner et al., 2013). The correlation coefficients were transformed into z-scores by Fisher’s z transformation method and then were exported into SPSS 18.0. Independent two-sample *t*-tests were used to detect group differences in FC. After that, false discovery rate (FDR, *p* < 0.05) corrections for multiple hypotheses testing were applied.

#### Brain-Behavior Analysis

Pearson correlation analyses were conducted to detect the correlation between z-scores of atypical FCs in CD adolescents within each network and corresponding empathy score. Pearson correlation analyses were performed using SPSS 18.0.0 in CD and HC group separately. False discovery rate corrections (FDR, *p* < 0.05) for multiple hypotheses testing were applied.

## Results

### Demographic and Behavioral Data

The demographic and psychiatric characteristics of the CD and HC groups are reported in Table 1. Age and IQ did not differ significantly between the two groups (both *p* > 0.05). Relative to the HC group, the CD group had a significant lower cognitive empathy score (*t* = -2.34, *p* = 0.023), a significant lower affective empathy score (*t* = -2.36, *p* = 0.022), and a significantly higher conduct problems trait score (*t* = 3.67, *p* = 0.001).

**Table 1:** Demographic and behavioral characteristics of the study cohort.

Characteristic	CD (*n* = 30)	HC (*n* = 33)	*t*	*p*
	Mean ± SD	Mean ± SD		
Age (years)	15.07 ± 0.52	15.27 ± 0.45	-1.68	0.098
IQ	105.71 ± 3.51	107.16 ± 3.29	-1.69	0.096
IRI total	21.20 ± 3.09	23.17 ± 2.32	-2.87	0.006
IRI-perspective taking	19.98 ± 4.50	22.76 ± 3.58	-2.72	0.008
IRI-empathic concern	21.31 ± 3.52	24.45 ± 3.81	-3.39	0.001
IRI-personal distress	21.18 ± 3.31	21.87 ± 5.24	-0.62	0.537
IRI-Fantasy	22.34 ± 5.04	23.59 ± 4.28	-1.06	0.294
Cognitive empathy score	21.16 ± 3.97	23.17 ± 2.79	-2.34	0.023
Affective empathy score	21.25 ± 2.76	23.17 ± 3.59	-2.36	0.022
SDQ-Conduct problems	4.02 ± 1.96	2.42 ± 1.48	3.67	0.001
APSD-Callous unemotional	5.93 ± 2.18	5.18 ± 1.69	1.54	0.129


*Two-sample t-test; IQ, intelligence quotient, CD, conduct disorder, HC, healthy controls, IRI, Interpersonal Reactivity Index Scale, SDQ, Strengths and Difficulties questionnaire, APSD, Antisocial Process Screening Device.*

### Functional Connectivities Within the Key Brain Regions of Cognitive Empathy

The group comparison results of analyses of covariance in FC involving the cognitive empathy network (vmPFC-dmPFC, vmPFC-rTPJ, vmPFC-lTPJ, vmPFC-lSTS, vmPFC-rSTS, dmPFC-rTPJ, dmPFC-lTPJ, dmPFC-lSTS, dmPFC-rSTS, rTPJ-lTPJ, rTPJ-lSTS, rTPJ-rSTS, lTPJ-lSTS, lTPJ-rSTS and lSTS-rSTS) are reported in Table 2 and Figure 1C. Notably, relative to the HC group, the CD group was found to have significantly weaker vmPFC-rTPJ (*t*_61_ = -3.012, *q_FDR_* = 0.017 ), and dmPFC-rTPJ FCs (*t*_61_ = -2.977, *q_FDR_* = 0.017).

**Table 2:** Group differences between the CD group and HCs within cognitive empathy network.

				*Uncorrected*
FC	CD (*n* = 30)	HC (*n* = 33)	*t*	*p-value*	*q_FDR_*
	Mean ± SD	Mean ± SD			
vmPFC-dmPFC	0.78 ± 0.28	0.83 ± 0.29	-0.729	0.469	0.395
vmPFC-rTPJ	0.43 ± 0.27	0.61 ± 0.21	-3.012	0.004	0.017
vmPFC-lTPJ	0.33 ± 0.31	0.44 ± 0.30	-1.495	0.140	0.242
vmPFC-lSTS	0.33 ± 0.26	0.41 ± 0.32	-1.102	0.275	0.339
vmPFC-rSTS	0.31 ± 0.28	0.31 ± 0.31	-0.002	0.999	0.575
dmPFC-rTPJ	0.50 ± 0.29	0.68 ± 0.20	-2.977	0.004	0.017
dmPFC-lTPJ	0.48 ± 0.31	0.45 ± 0.35	0.441	0.661	0.475
dmPFC-lSTS	0.35 ± 0.30	0.41 ± 0.29	-0.807	0.423	0.395
dmPFC-rSTS	0.44 ± 0.31	0.35 ± 0.35	1.015	0.314	0.339
rTPJ-lTPJ	0.39 ± 0.27	0.40 ± 0.30	-0.194	0.847	0.556
rTPJ-lSTS	0.33 ± 0.27	0.32 ± 0.21	0.124	0.902	0.556
rTPJ-rSTS	0.39 ± 0.35	0.31 ± 0.31	1.041	0.302	0.339
lTPJ-lSTS	0.62 ± 0.32	0.57 ± 0.32	0.673	0.504	0.395
lTPJ-rSTS	0.70 ± 0.27	0.56 ± 0.33	1.780	0.080	0.173
lSTS-rSTS	0.65 ± 0.30	0.52 ± 0.29	1.806	0.076	0.173


*FDR correction, FC, functional connectivity, CD, conduct disorder, HC, healthy controls, vmPFC, ventromedial prefrontal cortex, dmPFC, dorsomedial prefrontal cortex, TPJ, temporo-parietal junction, STS, superior temporal sulcus.*

### Functional Connectivities Within the Key Brain Regions of Affective Empathy

The group comparison results of analyses of covariance involving the affective empathy network (ACC-lIFG, ACC-rIFG, ACC-lAI, ACC-rAI, ACC-SM, lIFG-rIFG, lIFG-lAI, lIFG-rAI, lIFG-SM, rIFG-lAI, rIFG-rAI, rIFG-SM, lAI-rAI, lAI-SM, rAI-SM) are reported in Supplementary Table S2 and Figure 1D. There were no significant differences between the CD group and HC group in FCs within the affective empathy network.

### Brain-Behavior Analyses

There were no significant correlations between the z-scores of altered FCs within the key brain regions of cognitive empathy (vmPFC-rTPJ, *p* = 0.094; dmPFC-rTPJ, *p* = 0.151) and the behavioral score of cognitive empathy in CD adolescents. Besides, there were no significant correlations (vmPFC-rTPJ, *p* = 0.794; dmPFC-rTPJ, *p* = 0.876) were observed within the altered FCs and the cognitive empathy score.

## Discussion

The present study was the first to employ resting-state fMRI to explore FCs underlying affective and cognitive empathy in adolescents with CD. The behavioral analyses demonstrated that the CD adolescents had an affective and cognitive empathy deficit. In terms of the FC analyses, we found that CD adolescents exhibited decreased frontotemporal connectivity within the key brain regions of cognitive empathy, specifically, the vmPFC-rTPJ and dmPFC-rTPJ FCs. However, we did not find any evidence of an atypical FC within the key brain regions of affective empathy.

Our findings reflected that the CD adolescents had decreased FC between the mPFC and TPJ. The activity of both regions have been frequently reported in the empathic neural responses induced by observing painful pictures (Lamm et al., 2007, 2011). The TPJ is an association cortex that integrates input from the lateral and posterior thalamus, as well as from visual, auditory, somatosensory, and limbic areas (Decety and Lamm, 2007). The mPFC is a highly interconnected brain region with notable afferents from the dorsal-lateral prefrontal cortex, anterior STS, TPJ, and other brain regions (Van Overwalle, 2009). The considerable neural input that the mPFC receives may contribute to its capacity for abstract inference making (Leslie et al., 2004; Amodio and Frith, 2006; Van Overwalle, 2009). Generally, the TPJ has been linked with transient detection and evaluation of information of another’s state at a relatively perceptual level, whereas the mPFC has been associated with longer lasting processing of information related to the self and more abstract cognition (Gallagher and Frith, 2003; Shamay-Tsoory et al., 2009; Van Overwalle, 2009; Van Overwalle and Baetens, 2009; Raz et al., 2014). Appropriate medial-fronto-temporal communication is thought to facilitate the use of external social cues from temporal regions when deducing the internal emotional states of others in the mPFC (Abram et al., 2016). Given the mPFC, especially the vmPFC, is a crucial and specific brain region of the cognitive empathy (Shamay-Tsoory et al., 2006, 2009; Shamay-Tsoory and Aharon-Peretz, 2007), reduced connectivity between vmPFC and right TPJ in CD may reflect reduced access to external social cues in relation to cognitive empathy. Such a phenomenon may explain why adolescents with CD have poor empathic accuracy (Martin-Key et al., 2016).

Notably, we found that the z-scores of altered FCs within the key brain regions of cognitive empathy are not significantly positively associated with the cognitive empathy score measured by the IRI scale. Several possible reasons may contribute to these. On the one hand, distinct empathic neural responses may not necessarily result in different conscious subjective ratings indicated by the measures used in the present study. This might reflect the atypical FCs observed here was unconscious. Similar phenomenon has also been found in the task-fMRI studies which explored the empathy (Xu et al., 2009; Dong et al., 2016). On the other hand, the adolescents are prone to have some bias in fill out the self-report measures. Herpertz et al. (2010) also found that the neuroimaging data were not consistently with the self-report measures in CD adolescents, which may reflect that CD boy’s self-image of being “cool” rather than their real emotional experience. Overall, the empathy related behavior-brain correlations still need further explanations in the future studies.

Interestingly, we observed reduced vmPFC-TPJ FC only in the right hemisphere. The right TPJ has been described as playing a pivotal role in self-other discrimination (Decety and Lamm, 2006). Uddin et al. (2006) demonstrated selective impairment of self-other distinction when repetitive transcranial magnetic stimulation was applied over the right TPJ of participants performing a perceptual task involving discrimination between images of one’s own face and other familiar faces, thereby providing direct evidence for a casual role for this region in self-other discrimination. The findings of Decety and Sommerville (2003) further indicated that the right TPJ within the right fronto-parietal network played a pivotal role in distinguishing self from other, and that the prefrontal cortex was integral to coordinate and contrast cognitive representation of self and other. Hence, atypical FCs between the right TPJ and mPFC suggest that adolescents with CD may have a deficiency in self-other discrimination, a crucial component of cognitive empathy (de Waal, 2008; Singer and Lamm, 2009; Shamay-Tsoory, 2011).

With regard to the affective empathy, we found that the CD adolescents had lower affective empathy score, suggesting that the CD adolescents had a deficiency of affective empathy from the behavioral level. Previous task-fMRI studies (Decety et al., 2009; Lockwood et al., 2013; Schwenck et al., 2016) also found children with conduct problems exhibited atypical empathic neural responses in brain regions associated with affective empathy. Interestingly, we did not observe any atypical resting FC within the key brain regions of affective empathy in CD adolescents. Several reasons may account for this result. On the one hand, previous articles (Lamm et al., 2007; Bernhardt and Singer, 2012; Vera-Estay et al., 2016; Lockwood et al., 2017) have documented that the empathy is heavily context-dependent, being crucially affected by the agent’s motivation as well as the various social factors. Therefore, atypical affective neural responses by adolescents with CD, reported by the previous studies might have something to do with who they are feeling with and for, not the general malfunctioning. On the other hand, since many studies indicated that CU trait is an important factor influencing the affective empathy (Cheng et al., 2012; Schwenck et al., 2012), the matched-CU trait between the CD adolescents and HCs may also be the potential influential factor contributing to this result. Overall, further investigations, which explore the network alterations of affective empathy in CD adolescents with a task fMRI method and bigger sample, are necessary.

There are several limitations of this work that should be mentioned. First, the limited sample size can easily lead to false positives and inflated effected sizes (Yarkoni, 2010). Although the sample size in the present study is not less than similar studies typically done in this domain (Zhang et al., 2014; Jiang et al., 2016; Sun et al., 2018), the results in the present study still need a further verification with a bigger sample. Second, although ROI-based method has been frequently used in previous studies (Lockwood et al., 2013; Raz et al., 2014), it has an inherent methodological constraint: subjective ROIs selection. Besides, our study was only on the basis of resting fMRI data. Overall, the conclusion in the present study still needs further confirmation and replication of the task-based and experimental studies. Specifically, exploring the whole-brain FCs seeding in the crucial regions of cognitive and affective empathy is extremely meaningful whiling analyzing the empathy-related task-data. Third, the generalization of our result is still under restriction to some extent as we control the effect of CU trait. Therefore, our results still need further extensions in CD adolescents with and without CU trait.

## Conclusion

In conclusion, the present study represents the first attempt to investigate FCs related to affective and cognitive empathy in adolescents with CD. Reduced frontotemporal FCs (vmPFC/dmPFC-rTPJ) within the key brain regions of cognitive empathy were observed in adolescents with CD. Atypical FC within the key brain regions of affective empathy network was not observed in the CD group. Given that the vmPFC is a core region of social inference, and the TPJ is a primary region integrating the perceptual input, the observed decoupling between vmPFC/dmPFC and rTPJ in CD adolescents may reveal an insufficient use of external input and less involvement of social inference while empathizing with others. The present study may partly uncover the neural mechanism underlying the cognitive empathy deficiency in CD adolescents.

## Author Contributions

SY and XW designed the study. YJ, YG, QM, and DD performed the study. DD analyzed the data and wrote the manuscript.

## Conflict of Interest Statement

The authors declare that the research was conducted in the absence of any commercial or financial relationships that could be construed as a potential conflict of interest.

## Footnotes

1 http://www.restfmri.net
