# Supplementary material for: Atypical Frontotemporal Connectivity of Cognitive Empathy in Male Adolescents With Conduct Disorder
Source: Front Psychol. 2019 Jan 11;9:2778. doi: 10.3389/fpsyg.2018.02778 (PMC6338247; doi:10.3389/fpsyg.2018.02778)
Supplement: Supplementary file 1 [file Data_Sheet_1.PDF]

Supplementary materials

Table S1 Network ROIs and the MNI coordinates of their centers.

Brain region	***X***	***Y***	***Z***
Cognitive empathy network			
Ventromedial prefrontal cortex	0	60	18
Dorsomedial prefrontal cortex	2	58	30
Right temporoparietal junction	56	-48	24
Left temporoparietal junction	-50	-50	24
Right superior temporal sulcus	62	-32	0
Left superior temporal sulcus	62	-24	-10
Affective empathy network			
Medial/anterior cingulate cortex	6	18	30
Right anterior insula (dorsral)	39	12	15
Left anterior insula (ventral)	-33	3	-18
Left anterior insula\ inferior frontal gyrus	-45	15	-6
Right inferior frontal cortex	40	24	-4
Right supplementary motor cortex	6	8	58

Table S2 Group differences between the CD group and healthy controls in affective empathy network.

FC	CD (*n* = 30)	HC (*n* = 33)	*t*	*p*
	Mean ± SD	Mean ± SD		
ACC-lIFG	0.57 ± 0.30	0.61 ± 0.37	− 0.565	0.574
ACC-rIFG	0.58 ± 0.23	0.58 ± 0.31	− 0.073	0.942
ACC-lAI	0.55 ± 0.25	0.52 ± 0.32	0.414	0.680
ACC-rAI	0.43 ± 0.30	0.38 ± 0.23	0.838	0.405
ACC-rSM	0.75 ± 0.28	0.72 ± 0.32	0.382	0.704
lIFG-rIFG	0.71 ± 0.28	0.64 ± 0.39	0.755	0.453
lIFG-lAI	0.48 ± 0.29	0.41 ± 0.34	0.866	0.390
lIFG-rAI	0.29 ± 0.26	0.26 ± 0.30	0.428	0.670
lIFG-rSM	0.61 ± 0.33	0.56 ± 0.32	0.574	0.568
rIFG-lAI	0.42 ± 0.23	0.42 ± 0.29	− 0.013	0.989
rIFG-rAI	0.40 ± 0.26	0.42 ± 0.29	1.167	0.248
rIFG-rSM	0.55 ± 0.24	0.55 ± 0.27	− 0.012	0.991
lAI-rAI	0.28 ± 0.21	0.22 ± 0.19	1.204	0.233
lAI-rSM	0.41 ± 0.23	0.40 ± 0.28	0.227	0.821
rAI-rSM	0.36 ± 0.31	0.33 ± 0.23	0.367	0.715

Note. CD, conduct disorder; HC, healthy controls; ACC, anterior cingulate cortex; IFG, inferior frontal cortex; AI, anterior insula; SM, supplementary motor area.
